# Distinct Distribution of RTN1A in Immune Cells in Mouse Skin and Lymphoid Organs

**DOI:** 10.3389/fcell.2020.608876

**Published:** 2021-01-15

**Authors:** Małgorzata Anna Cichoń, Katharina Klas, Maria Buchberger, Martina Hammer, Kristin Seré, Martin Zenke, Erwin Tschachler, Adelheid Elbe-Bürger

**Affiliations:** ^1^Department of Dermatology, Medical University of Vienna, Vienna, Austria; ^2^Institute of Cancer Research, Medical University of Vienna, Vienna, Austria; ^3^Department of Cell Biology, Institute for Biomedical Engineering, RWTH Aachen University Medical School, Aachen, Germany; ^4^Helmholtz Institute for Biomedical Engineering, RWTH Aachen University, Aachen, Germany

**Keywords:** RTN1A, Langerhans cell, dendritic cell, immune cell, skin, lymphoid organ, nerves, endoplasmic reticulum

## Abstract

The endoplasmic reticulum-associated protein reticulon 1A (RTN1A) is primarily expressed in neuronal tissues but was recently identified also specifically in cells of the dendritic cell (DC) lineage, including epidermal Langerhans cells (LCs) and dermal DCs in human skin. In this study, we found that in mice major histocompatibility complex class II (MHCII)^+^CD207^+^ LCs but not dermal DCs express RTN1A. Further, RTN1A expression was identified in CD45^+^MHCII^+^CD207^+^ cells of the lymph node and spleen but not in the thymus. Of note, RTN1A was expressed in CD207^*low*^ LCs in adult skin as well as emigrated LCs and DCs in lymph nodes and marginally in CD207^*hi*^ cells. Ontogeny studies revealed that RTN1A expression occurred before the appearance of the LC markers MHCII and CD207 in LC precursors, while dermal DC and T cell precursors remained negative during skin development. Analogous to the expression of RTN1A in neural tissue, we identified expression of RTN1A in skin nerves. Immunostaining revealed co-localization of RTN1A with nerve neurofilaments only in fetal but not in newborn or adult dermis. Our findings suggest that RTN1A might be involved in the LC differentiation process given its early expression in LC precursors and stable expression onward. Further analysis of the RTN1A expression pattern will enable the elucidation of the functional roles of RTN1A in both the immune and the nervous system of the skin.

## Highlights

- In adult mouse skin RTN1A is exclusively expressed in resident and migrating Langerhans cells (LCs).
- RTN1A expression is associated with MHCII^+^CD207^*lo*^ LCs/Dendritic cells (DCs) rather than MHCII^+^CD207^*hi*^ LCs/DCs.
- RTN1A expression is enriched in the dendrites of LCs in the skin and DCs in primary and secondary lymphoid organs.
- LC precursors and cutaneous nerves express RTN1A in prenatal skin.

## Introduction

The endoplasmic reticulum (ER) is the largest and most spatial organelle in mammalian cells (Baumann and Walz, 2001) comprising a nuclear envelope and the peripheral ER with rough and smooth membrane domains (Shibata et al., 2006). The smooth ER (SER) is an extensive network of highly curved membrane tubules outstretching through the whole cytoplasm of the cell (Westrate et al., 2015). Reticulon (RTN) proteins are integral membrane proteins expressed in the tubules of the SER network and are engaged in tubule membrane curvature (Voeltz et al., 2006; Westrate et al., 2015). Alternative splicing of RTN genes results in several protein isoforms (RTN1-4, A-C) (Yang and Strittmatter, 2007). All RTNs have a reticulon homology domain (RHD), an evolutionary conserved region that is the same for all RTN family members across all eukaryotes. The RHD at the C-terminal domain contains two intramembrane hydrophobic segments, separated by a hydrophilic loop (Yang and Strittmatter, 2007). The RHD is not only involved in the ER curvature but also in selective ER-phagy (Bhaskara et al., 2019). Conversely, the N-terminal region varies functionally between paralogs and because of its length can be divided into long (e.g., isoform A) and short (e.g., isoform C) forms (Chiurchiù et al., 2014; Grumati et al., 2017). Both, N- and C-terminal domains are directed toward the intracellular space (Voeltz et al., 2006; Zurek et al., 2011). Although biological functions and roles are identified for many proteins, knowledge about alternative spliced isoforms of a particular protein is often sparse. This includes whether the isoform (i) is expressed, and if so, in what cell type and at what stage of development, (ii) is altered with disease or (iii) has distinct cell localization and function (Stastna and Van Eyk, 2012).

The RTN1A isoform was initially identified in neuroendocrine tissue (Roebroek et al., 1993; Stastna and Van Eyk, 2012) and was subsequently shown to be essential for ER-mitochondrial contact formation (Cho et al., 2017), and controlling of calcium flux in neurons via inhibition of calcium release from the ER store (Kaya et al., 2013). Furthermore, RTN1p, a yeast RTN1A ortholog, promotes SER tubule curvature (Hu et al., 2008). Interestingly, overexpression of human RTN1A in mice causes ER stress-related apoptosis, while a specific knockdown of RTN1A in renal tubular epithelial cells in mice reduces ER stress and renal fibrosis (Fan et al., 2017).

Barrier tissues are the first defense mechanism, protecting human body from environmental stressors. A well-functioning network of antigen-presenting cells (APCs) is crucial for detecting pathogens penetrating the skin. In APCs, SER is also essential for antigen processing and presentation (Kotsias et al., 2019). Recently, our group identified RTN1A expression in prenatal and adult human skin exclusively in epidermal Langerhans cells (LCs) and dermal dendritic cells (DCs) and suggested it as a marker specific for cutaneous DCs (Gschwandtner et al., 2018). Here we set out to analyze the expression of RTN1A gene and protein in murine skin and lymphoid tissues and found that its expression is restricted to subsets of APCs but absent from other immune cells.

## Results

### RTN1A Expression Is Specific for Resident and Migrating LCs in Adult Mouse Skin

**FIGURE 1:**
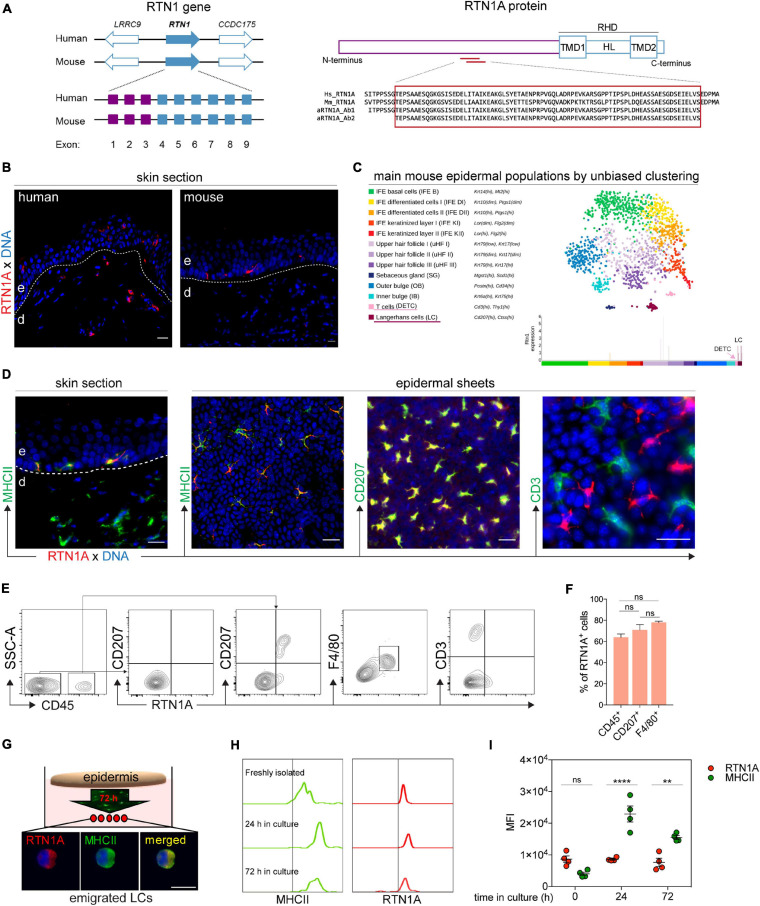
RTN1A expression is specific for resident and migrating LCs in adult mouse skin. **(A)** Scheme of the RTN1 gene with flanking genes in human and mouse; purple boxes indicate exons coding for 1A isoform and blue boxes for RHD. Following the linear structure of the RTN1A protein with αRTN1A antibodies binding sites specific in both species. TMD-transmembrane domains, HL-hydrophilic loop, RHD-reticulon homology domain. **(B)** Representative RTN1A IF staining of adult human and mouse skin sections shows differences in its expression pattern in cutaneous DCs, *n* = 3, e-epidermis, d-dermis, scale bar: 20 μm. **(C)** Single cell RNA-sequencing map (t-SNE plot) with mouse epidermal cell populations using unbiased clustering. The RTN1 gene is expressed in the LC but not DETC cluster. **(D)** Representative IF staining of adult mouse skin sections identifies MHCII^+^RTN1A^+^ LCs in the epidermis and MHCII^+^RTN1A^–^ DCs in the dermis. Epidermal sheet staining unraveling RTN1A co-expression with MHCII and CD207 but not CD3. *n* = 3, scale bar: 20 μm. **(E)** Representative contour plots of epidermal single cell suspensions from back skin measured by flow cytometry. Living-singlets were gated for markers including CD45 (leukocytes), CD3 (T cells), CD207 and F4/80 (LCs). *n* = 3. **(F)** Bar graph showing percentage of RTN1A^+^ cells in epidermis from back skin co-expressing CD45, CD207, and F4/80, respectively. Data was obtained from flow cytometry analysis and are shown as standard error of the mean (SEM) from 3 independent experiments. Statistical analysis of the data has been performed using One-way ANOVA, multiple comparisons (Tukey’s multiple comparisons test): ns, *p* > 0.05. ns- not significant. **(G)** Epidermal sheets from adult mouse ears were cultured for 72 h for LC emigration. Shown is one representative IF staining of emigrated LCs on adhesion slides co-expressing RTN1A and MHCII. *n* = 3, scale bar: 20 μm. **(H,I)** Comparative assessment of RTN1A and MHCII expression in immature and emigrated LCs using flow cytometry. Representative histograms **(H)** and quantitative MFI evaluation plot **(I)** showing RTN1A and MHCII expression in LCs of freshly isolated ear epidermal sheets and upon *in vitro* culture after 24 and 72 h. Statistical analysis of the data has been performed using One-way ANOVA, multiple comparisons (Tukey’s multiple comparisons test): ns, *p* > 0.05; **p ≤ 0.01; ****p ≤ 0.0001. MFI-mean fluorescent intensity, ns- not significant, *n* = 4.

In our previous study we reported that RTN1A protein is abundantly expressed in epidermal LCs, dermal DCs and their precursors in human skin (Gschwandtner et al., 2018). Based on these findings we next characterized the RTN1A expression profile in cutaneous mouse APCs. The RTN1A protein is encoded by the RTN1 gene that is located in the same locus in human and mouse with mainly the same flanking genes (Figure 1A). Moreover, in both species exons 1–3 encode the long RTN isoform 1A and exons 4–9 the RHD (Figure 1A and Supplementary Table 1). Protein sequence alignment revealed that RTN1A is highly conserved between human and mouse (Supplementary Figure 1A). Additionally, antibodies specific for human RTN1A are binding with the same specificity to RTN1A in mice (αRTN1A epitopes do not overlap neither with the isoform 1C nor with the RHD region) (Figure 1A and Supplementary Figures 1A,B). In contrast to the RTN1A expression profile in human skin, IF labeling of adult mouse ear skin sections revealed strong positive signals restricted to epidermal dendritically shaped cells (Figure 1B). Single cell transcriptome analysis of mouse epidermis showed RTN1 gene expression exclusively in LCs, and that its expression was lower compared to that of typical LC markers such as MHCII and CD207 (Figure 1C and Supplementary Figures 2A–E). Double IF staining of skin sections and epidermal sheets of adult mice confirmed RTN1A co-expression in MHCII^+^CD207^+^ LCs but not in dendritic epidermal T cells (DETCs) (Figure 1D). A continuum expression of RTN1A^*lo*^ to RTN1A^*hi*^ was observed in LCs *in situ*. Of note, RTN1A staining was particularly strong in dendrites as compared to the cell body (Figure 1D). Next, epidermis from back skin was enzymatically separated from the dermis and the epidermal single cell suspension was gated for CD45^+^ leukocytes and CD45^–^ cells using flow cytometry. Confirming and extending our observations *in situ*, we found that within CD45^+^ leukocytes the majority was CD207^*lo*^RTN1A^*hi*^, while the minority was CD207^*hi*^RTN1A^*lo*^, indicative for different LC maturation stages (Figure 1E). Employment of another well-established LC marker (=F4/80) (Larsen et al., 1990; Ginhoux et al., 2006) confirmed the LC nature of CD207^*lo*^RTN1A^*hi*^ cells (Figure 1E). Quantification of RTN1A^+^ cells co-expressing CD45, CD207, and F4/80 showed that not all CD207^+^ and F4/80^+^ LCs express RTN1A (Figure 1F), which is in line with our previous observation in human skin (Gschwandtner et al., 2018). In average, 70% of CD207^+^ LCs and 77.8% of F4/80^+^ LCs expressed RTN1A (Table 1). The difference between co-expression of these two markers was not significant. In contrast, CD45^+^CD3^+^ DETCs did not express RTN1A at the gene (Figure 1C) and protein level (Figures 1D,E). The analysis of dermal cell suspensions repeatedly showed a minor CD45^+^RTN1A^+^ population, most likely representing migratory LCs (Supplementary Figure 2F).

**TABLE 1:** RTN1A^+^ cells co-expressing CD45, CD207, and F4/80 in skin and lymphoid organs in adult mice.

	RTN1A co-expression with immune cell markers		
Tissue	CD45	CD207	F4/80
skin	63.8%	70.8%	77.8%
thymus	1.5%	0.1%	0.8%
lymph node	19.0%	29.0%	76.0%
spleen	11.3%	80.0%	60.7%

*Data were analyzed by flow cytometry and are shown as mean from 3 independent experiments. Shown are percentages of RTN1A^+^ cells in CD45 pre-gated cell populations.*

Upon antigen stimulation, LCs migrate out of the epidermis and regulate the expression of specific markers (e.g., E-cadherin, MHCII, co-stimulatory, and activation molecules etc.) (Clausen and Stoitzner, 2015). To explore whether RTN1A is similarly regulated during LC migration and since RTN1A has been described to be involved in the migration of bone marrow-derived macrophages toward a chemoattractant (McGonigle et al., 2018), we comparatively assessed its expression levels in freshly isolated immature LCs and emigrated LCs upon culture of ear epidermal sheets (Figure 1G). Mean fluorescence intensity values, as measured by flow cytometry, showed that MHCII expression was significantly upregulated after 24 and 72 h compared with freshly isolated LCs, while RTN1A expression remained stable during LC migration out of cultured epidermal sheets (Figures 1H,I). Assessment of emigrated LCs upon 72 h of culture revealed a roundish morphology and co-expression of RTN1A and MHCII (Figure 1G). Together, our data show that in the mouse skin only LCs express RTN1A and that its expression intensity is not regulated upon migration and culture. Thus, RTN1A can be employed as a reliable LC marker in future studies.

### LC Precursors and Nerves in Developing Skin Express RTN1A

**FIGURE 2:**
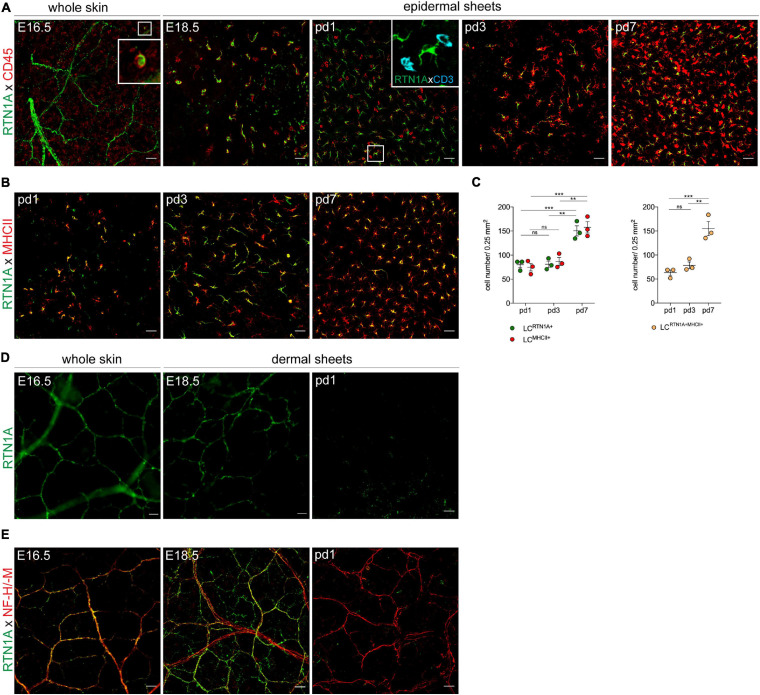
LC precursors and nerves express RTN1A in prenatal mouse skin. **(A)** Representative IF staining of RTN1A and CD45 (leukocytes) in whole embryonic back skin (E16.5) and in prenatal (E18.5) and postnatal (pd1, 3, 7) epidermal sheets from back skin. Zoom-in of boxed area at E16.5. shows one RTN1A^+^CD45^+^ cell. Kinetic experiments revealed that CD45^+^CD3^–^ cells (=LC precursors) but not CD45^+^CD3^+^ (=DETC precursors, pd1, insert, azure) express RTN1A. *n* = 3 per age group, E-embryonic day, pd-postnatal day, scale bars: 20 μm. **(B)** Representative IF staining of RTN1A and MHCII in postnatal (pd1, 3, 7) epidermal sheets from back skin. After birth, most RTN1A^+^ cells start to upregulate MHCII. *n* = 3 per age group, E-embryonic day, pd-postnatal day, scale bars: 20 μm. **(C)** Scatter dot blot showing quantified number of LCs expressing RTN1A or/and MHCII in epidermal sheets. Data are shown as standard error of the mean (SEM) from 3 independent experiments with three different donors. Statistical analysis of the data has been performed using 2-way ANOVA, multiple comparisons (Tukey’s multiple comparisons test): ns, *p* > 0.05, **p ≤ 0.01, ***p ≤ 0.001. **(D)** Representative IF staining of the RTN1A expression pattern in whole embryonic back skin (E16.5) and in dermal sheets from back skin (E18.5, pd1). RTN1A expression is not detectable in postnatal dermis. *n* = 3 per each age group. **(E)** Representative IF staining of RTN1A co-expression with an NF-M/-H nerve marker. RTN1A was detectable only in the prenatal dermis. **(D,E)** *n* = 3 per each age group. E-embryonic day, pd-postnatal day, scale bars: 20 μm.

Our observation that RTN1A is among the earliest markers to be expressed during LC differentiation in human embryonic skin (Gschwandtner et al., 2018), might suggest its involvement in LC differentiation and network formation. To test whether the expression pattern of RTN1A in developing mouse skin is comparable to humans, we employed IF labeling and confocal microscopy of whole skin sheets at embryonic day 16.5 (E16.5) as enzymatic separation of skin compartments is impossible yet, and epidermal/dermal sheets at E18.5 and postnatal days 1–7 (pd1-7) (Elbe et al., 1989). For the identification of leukocytes, RTN1A was co-stained with CD45. In whole skin sheets we found a few round to polygonal CD45^+^RTN1A^+^ cells (Figure 2A, E16.5, insert), and CD45^–^RTN1A^+^ string-like structures resembling skin nerves (Figure 2A, E16.5). Assessment of epidermal sheets at later time points of gestation revealed the presence of two distinct populations: a large population of polygonal to dendritic CD45^+^RTN1A^+^ cells and a small population of round CD45^+^RTN1A^–^ cells. Over the course of 1 week after birth, numbers of round CD45^+^RTN1A^–^ cells increased, acquired CD3 and became dendritic over time representing a population described to be DETCs (Figure 2A, pd1, insert) (Elbe et al., 1989). Further, a continuum expression of RTN1A^*lo*^ to RTN1A^*hi*^ on LCs during the observation period of pd1-7 has been observed (Figure 2B). Enumeration revealed a comparable number of RTN1A^+^ and MHCII^+^ cells which significantly increased during the first week after birth (Figure 2C), as described previously for MHCII^+^ LCs (Elbe et al., 1989). Expression of RTN1A in differentiating LCs throughout skin development before the expression of typical LC markers could suggest its involvement in LC differentiation also in mouse skin.

Analogous to the expression of RTN1A in human brain neurons (Roebroek et al., 1993; Gschwandtner et al., 2018), we identified RTN1A^+^ Purkinje cells in the mouse cerebellum (Supplementary Figure 3), thus confirming the specificity of antibodies used in this study. To test, whether RTN1A^+^ structures found in the prenatal skin (Figure 2A, E16.5) could represent cutaneous nerves, we performed RTN1A expression kinetic experiments with whole skin and dermal sheets from back skin. Again, we found an RTN1A staining pattern reminiscent of nerve structures in prenatal skin (E16.5, E18.5) that was undetectable 1 day after birth (pd1) (Figure 2D). Counterstaining of RTN1A^+^ structures with a marker detecting the middle (M) and heavy (H) subunits of neurofilaments (NF) (Yuan et al., 2017), revealed co-expression of the two markers only during early skin development as it was undetectable in skin after birth and not detectable in skin of adult mice (Figure 2E, and data not shown). In contrast to the lost expression of RTN1A after birth, the NF-M/H is still abundantly expressed (Figure 2E). These data suggest that RTN1A might be involved not only in the differentiation of LC precursors but also in cutaneous innervation in the developing skin.

### Distinct APCs Express RTN1A in Lymphoid Organs

**FIGURE 3:**
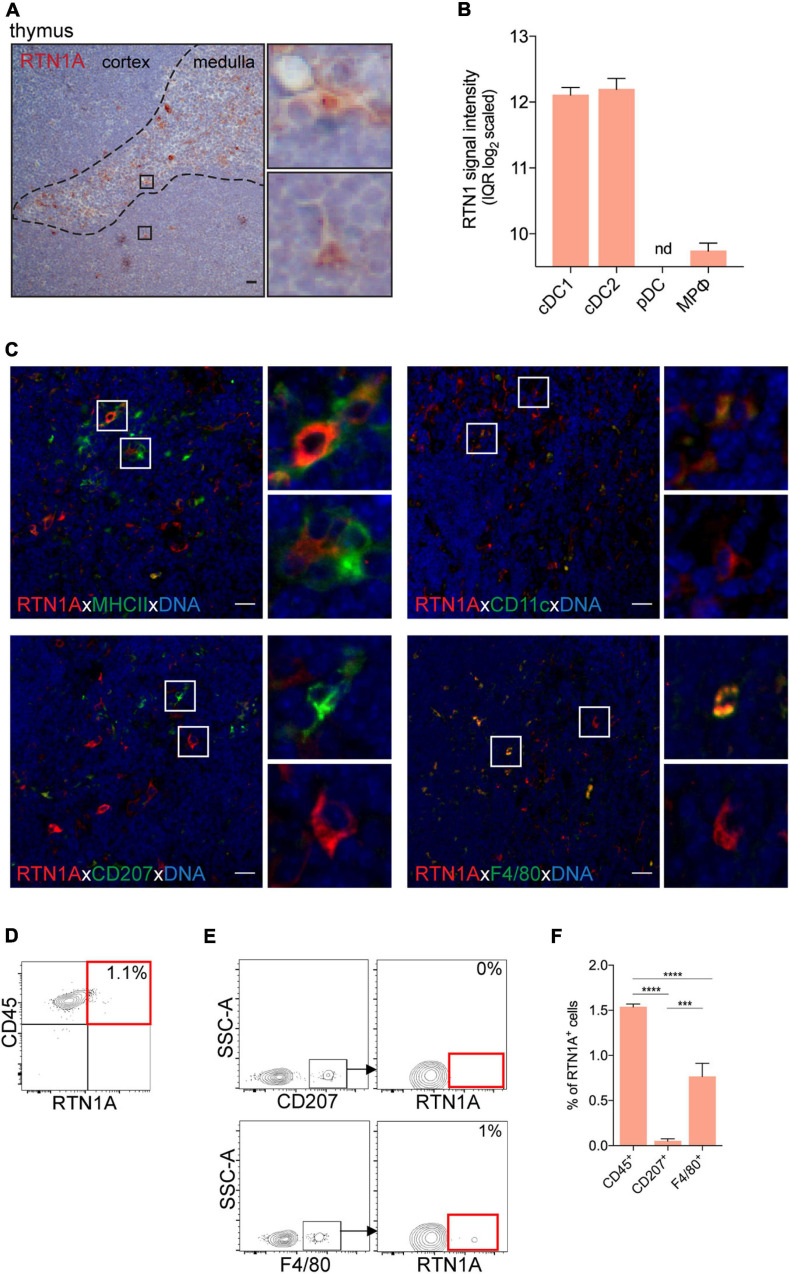
RTN1A expressing cells are mainly located in the thymic medulla of adult mice. **(A)** Representative RTN1A IHC staining of the thymus. Zoom-in of boxed areas shows RTN1A^+^ cells in thymic medulla and cortex. *n* = 3, scale bar: 20 μm. **(B)** RTN1 mRNA expression in indicated thymic immune cell types is shown as mean of RTN1 signal intensity (IQR log_2_ scaled). Data are retrieved from Genevestigator. Detailed flow cytometry gating strategy for all cell types, number of samples and experiment ID can be found in Supplementary Table 3. cDC-conventional dendritic cell, pDC-plasmacytoid dendritic cell, MPφ-macrophages, nd-not detected. **(C)** Representative IF staining of RTN1A co-expression with indicated APC markers. Zoom-in of boxed areas show double positive (upper pictures) and RTN1A single positive cells (lower pictures), except of CD207 staining where no double positive cells were detected. *n* = 4, scale bar: 20 μm. **(D,E)** Living singlets of thymic cell suspensions were gated on CD45 and RTN1A as well as F4/80/CD207 by flow cytometry. Percentages of RTN1A^+^ cells are indicated from one representative experiment. **(D)** *n* = 6 **(E)** *n* = 3. **(F)** Bar graph showing percentage of RTN1A^+^ cells in the thymus co-expressing CD45, CD207, and F4/80. Data were obtained from flow cytometry analysis and are shown as standard error of mean (SEM) from 3 independent experiments. Statistical analysis of the data has been performed using One-way ANOVA, multiple comparisons (Tukey’s multiple comparisons test): ***p ≤ 0.001; ****p ≤ 0.0001.

**FIGURE 4:**
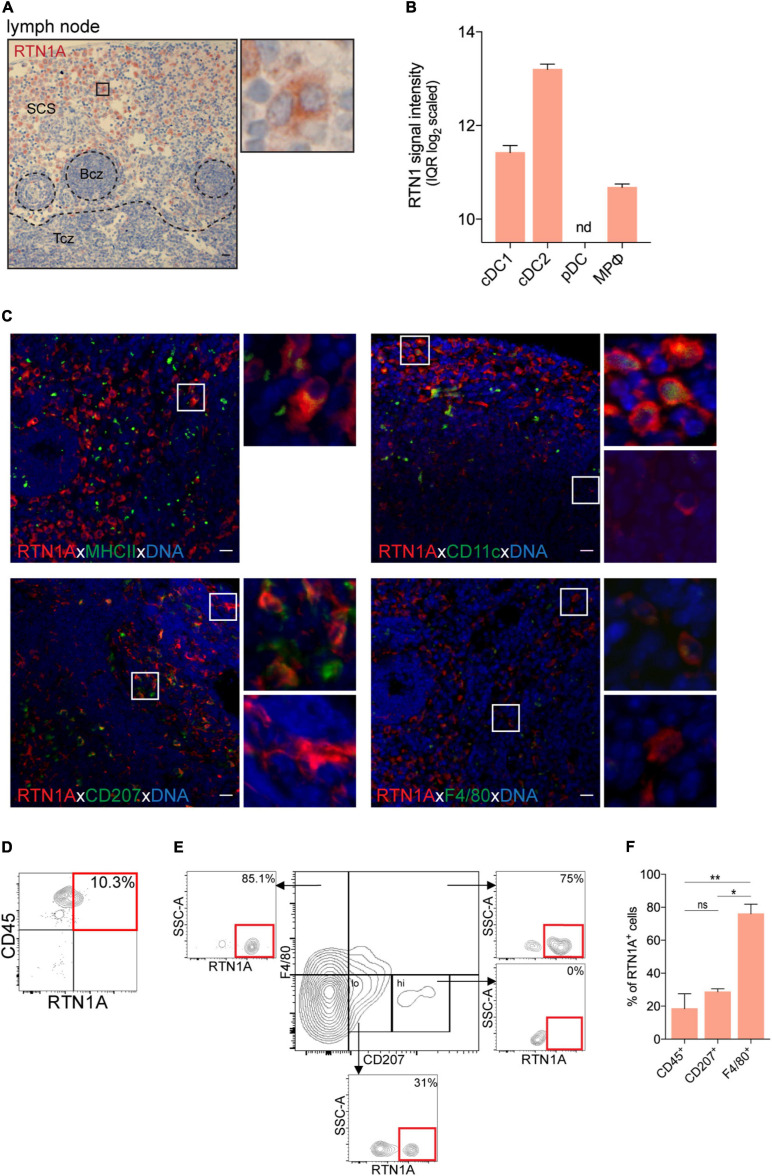
RTN1A^+^ cells are abundantly expressed in the subcapsular sinus of lymph nodes. **(A)** Representative RTN1A IHC staining of lymph node. Zoom-in of boxed area shows RTN1A^+^ cells in the subcapsular sinus. SCS-subcapsular sinus, Bcz-B cell zone, Tcz-T cell zone. *n* = 3, scale bar: 20 μm. **(B)** RTN1 mRNA expression in immune cell types in lymph node is shown as mean of RTN1 signal intensity (IQR log_2_ scaled). RTN1 gene expression levels in mouse immune cells are retrieved from Genevestigator. Detailed flow cytometry gating strategy for all cell types, number of samples and experiment ID can be found in Supplementary Table 3. cDC-conventional dendritic cell, pDC-plasmacytoid dendritic cell, MPφ-macrophages, nd-not detected. **(C)** Representative IF staining of RTN1A co-expression with APC markers. Zoom-in of boxed area shows double positive (upper pictures) and RTN1A single positive cells (lower pictures). *n* = 3, scale bar: 20 μm. **(D,E)** Living singlets were gated on CD45 and RTN1A. Subsequently, the CD45^+^ population was gated on F4/80/CD207 and RTN1A. Percentages of RTN1A^+^ cells are indicated from one representative experiment. **(D)** *n* = 5 **(E)** *n* = 3. **(F)** Bar graph showing percentage of RTN1A^+^ cells in inguinal lymph node co-expressing CD45, CD207, and F4/80, respectively. Data were obtained from flow cytometry analysis and shown as standard error of mean (SEM) from 3 independent experiments. Statistical analysis of the data has been performed using One-way ANOVA, multiple comparisons (Tukey’s multiple comparisons test): ns, *p* > 0.05; *p ≤ 0.05; **p ≤ 0,01. ns- not significant.

**FIGURE 5:**
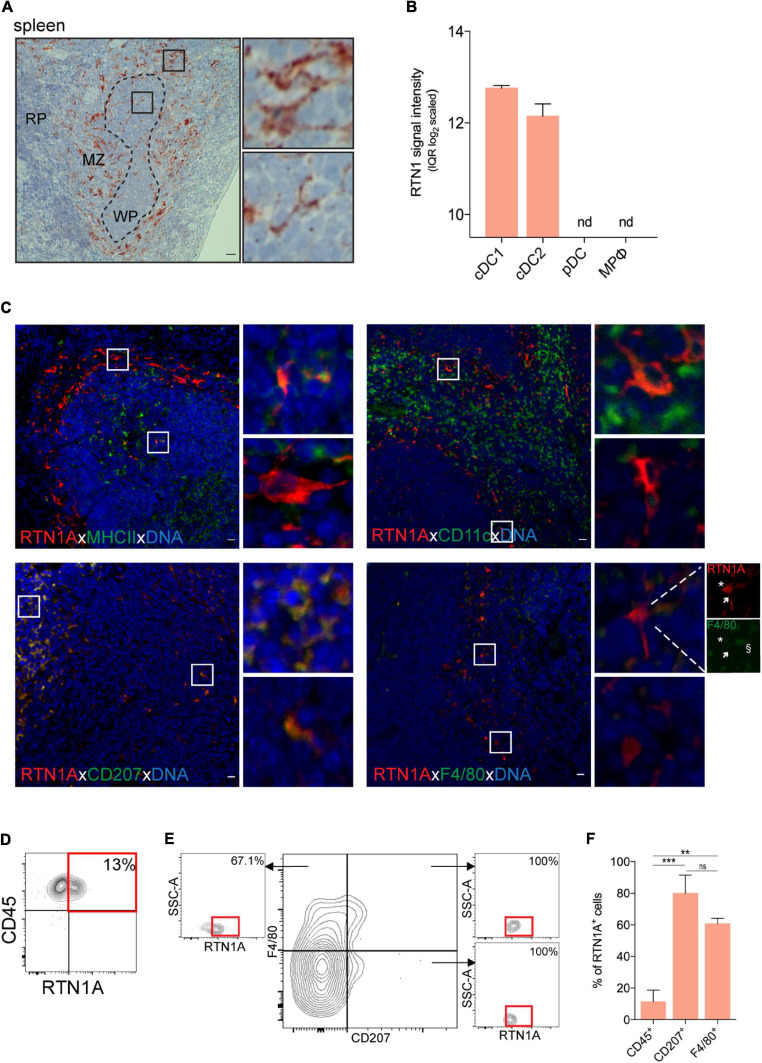
RTN1A expressing cells are predominantly localized in the splenic marginal zone. **(A)** Representative RTN1A IHC staining of spleen. Zoom-in of boxed areas shows RTN1A^+^ cells in marginal zone. MZ-marginal zone, WP-white pulp, RP-red pulp, scale bar: 20 μm. **(B)** RTN1 mRNA expression in immune cell types in spleen is shown as mean of RTN1 signal intensity (IQR log_2_ scaled). RTN1 gene expression levels in mouse immune cells are retrieved form Genevestigator. Detailed flow cytometry gating strategy for all cell types, number of samples and experiment ID can be found in Supplementary Table 3. cDC-conventional dendritic cell, pDC-plasmacytoid dendritic cell, MPφ-macrophages, nd-not detected. **(C)** Representative IF staining of RTN1A co-expression with APC markers. Zoom-in of boxed areas shows double positive (upper pictures) and RTN1A single positive cells (lower pictures). scale bar: 20 μm. **(D,E)** Living singlets were gated on CD45 and RTN1A, Subsequently, the CD45^+^ population was gated on F4/80/CD207 and RTN1A. Percentages of RTN1A^+^ cells are indicated from one representative experiment. **(D)** *n* = 6 **(E)** *n* = 3. **(F)** Bar graph showing percentage of RTN1A^+^ cells in spleen co-expressing CD45, CD207, and F4/80, respectively. Data were obtained from flow cytometry analysis and shown as standard error of mean (SEM) from 3 independent experiments. Statistical analysis of the data has been performed using One-way ANOVA, multiple comparisons (Tukey’s multiple comparisons test): ns- *p* > 0.05; **p ≤ 0.01; ***p ≤ 0.001. ns- not significant.

Peripheral lymphoid organs comprise a heterogeneous group of immune and non-immune cells which govern tissue homeostasis and are involved in immune responses (Mebius and Kraal, 2005). We analyzed and described the expression profile of RTN1A in immune cells in primary and secondary lymphoid organs. Using Genevestigator and Affymetrix microarray we analyzed mRNA expression of RTN1 in selected immune cell types of the thymus, lymph nodes and spleen (Supplementary Table 3). Further, we performed IHC and IF labeling of tissue sections to depict localization, distribution and morphology of RTN1A^+^ cells. In addition, we employed flow cytometry analysis of single cell suspensions to enumerate RTN1A^+^ leukocytes co-expressing CD207 and F4/80 in lymphoid organs comparable to our analyses of RTN1A^+^ cells in the epidermis. Of note, we found common features in the RTN1A protein expression profile throughout investigated organs as we identified RTN1A^+^ cells in areas typical for the occurrence of APCs such as the thymic medulla, subcapsular sinus in lymph nodes and splenic marginal zone (Figures 3A–5A). Furthermore, cells expressing RTN1A were also distinguishable from other cells by a larger size, dendritic morphology and distribution of this protein in cell dendrites or protrusions (Figures 3–5). The CD45 marker has been used to confirm the leukocyte lineage (Figures 3D–5D). T and B cells did not express RTN1A neither on protein (Supplementary Figures 3B,C) nor mRNA level (Supplementary Table 3).

The main DC type in the thymus are conventional DCs (cDCs) (Blackburn and Manley, 2004). mRNA analysis of RTN1 in the thymus revealed high expression levels in cDC1 (CD8α^+^) and cDC2 (CD8α^–^), while low or undetectable levels were observed in macrophages and plasmacytoid DCs (pDCs), respectively (Figure 3B). In IHC images, we have detected a small population of roundish and dendritic RTN1A^+^ cells primarily in the thymic medulla (Figure 3A). Double IF staining uncovered co-expression of RTN1A mainly with APC markers such as MHCII and CD11c (Figure 3C, upper panel), which confirms the results of mRNA analysis that cDC1 and cDC2 express RTN1. Further, only a few F4/80^+^ cells and no CD207^+^ cells co-expressed RTN1A (Figure 3C, lower panel). We confirmed these observations with flow cytometry, and showed that RTN1A is not expressed in CD207^+^ cells but cDCs in the thymus (Figures 3E,F) and that the RTN1A^+^F4/80^+^ (0.8%) cells were most likely macrophages found also by mRNA analysis (Figures 3E,F). The difference in RTN1A distribution and co-expression between analyzed markers (CD45, CD207, and F4/80) was significant (Figure 3F).

Skin draining lymph nodes are a reservoir of a heterogeneous group of immune cells. Together with tissue-migrating APCs, DCs and sinus-resident macrophages in subcapsular sinus of lymph nodes are considered as a frontline of immune protection (Louie and Liao, 2019). mRNA analysis of RTN1 expression showed that cDC2 (CD4^+^) expressed the highest RTN1 levels when compared with cDC1 (CD8α^+^) and macrophages, while pDCs were negative (Figure 4B). We found that in the lymph nodes the majority of RTN1A^+^ cells were localized in the subcapsular sinus region also surrounding B and T cell zones (Figure 4A). *In situ* staining revealed that the majority of MHCII^+^ and CD11c^+^ cells in the sinus co-expressed RTN1A^+^ (Figure 4C, upper panel), again confirming mRNA data for RTN1 expression in DCs. Furthermore, investigations of LCs with the CD207 marker, three populations were found: CD207^+^RTN1A^+^, CD207^–^RTN1A^+^, and CD207^+^RTN1A^–^ cells in the lymph node sinus (Figure 4C, lower panel). This observation was substantiated by flow cytometry showing that 31% of CD207^*lo*^ cells expressed RTN1A while CD207^*hi*^ cells were RTN1A^–^. The majority of CD207^+^F4/80^+^ cells (75%) and CD207^–^F4/80^+^ cells (85%) co-expressed RTN1A (Figure 4E). The percentages of RTN1A^+^ cells shown in brackets are from one representative flow cytometry analysis, while a detailed summary of RTN1A co-expression with single markers is provided in Figure 4F. Of note, as Langerin/CD207 expression is changing upon LC maturation and migration and this protein can be found also on DC subsets (Henri et al., 2010) we analyzed RTN1A expression in DC subtypes in inguinal lymph node via flow cytometry. We found that the overwhelming majority (89.7%) of LCs/DCs^*CD*207+CD103–^ expressed RTN1A. Furthermore, around 50% of cDC1^*CD*207+^ and cDC2 were RTN1A^+^, while cDC1^*CD*207–^ and pDCs were RTN1A negative (Supplementary Figures 4A,B).

Spleen is the largest secondary lymphoid organ in the body. The marginal zone (DCs, macrophages, innate immune cells) connects the white pulp (T and B cells) with the red pulp (mainly fibroblasts, reticular fibers and certain types of macrophages) (Wu et al., 2017; Backer et al., 2019). Analysis of mRNA data revealed that cDC1 (CD8α^+^) and cDC2 (CD4^+^) but neither pDCs nor macrophages express RTN1. In IHC-stained spleen sections we found that RTN1A was mainly expressed in the marginal zone (Figure 5A). Double IF labeling of sections uncovered a minute population of MHCII^+^RTN1A^+^ and CD11c^+^RTN1A^+^ cells (Figure 5C, upper panel), representing cDC1 and cDC2 subsets. MHCII^+^RTN1A^+^ and several RTN1A^+^CD207^+^ cells were localized mainly in the white pulp, where DCs were described to interact with T cells (Backer et al., 2019).

Of note, F4/80^*lo*^RTN1A^*hi*^ cells were more dendritic than F4/80^*hi*^RTN1A^*lo*^ cells (Figure 5C, lower panel, inserts). Flow cytometry analysis showed that all CD207^+^F4/80^+^ and CD207^+^F4/80^–^ cells were positive for RTN1A, and that the majority of CD207^–^F4/80^+^ cells (67%) expressed RTN1A (Figure 5E). The summary of double positive populations can be found in Figure 5F, where we compared RTN1A co-expression with CD45, CD207, and F4/80 and have shown that there is significant difference between these populations, except of CD207 and F4/80, where the difference was not significant (Figure 5E).

## Discussion

**FIGURE 6:**
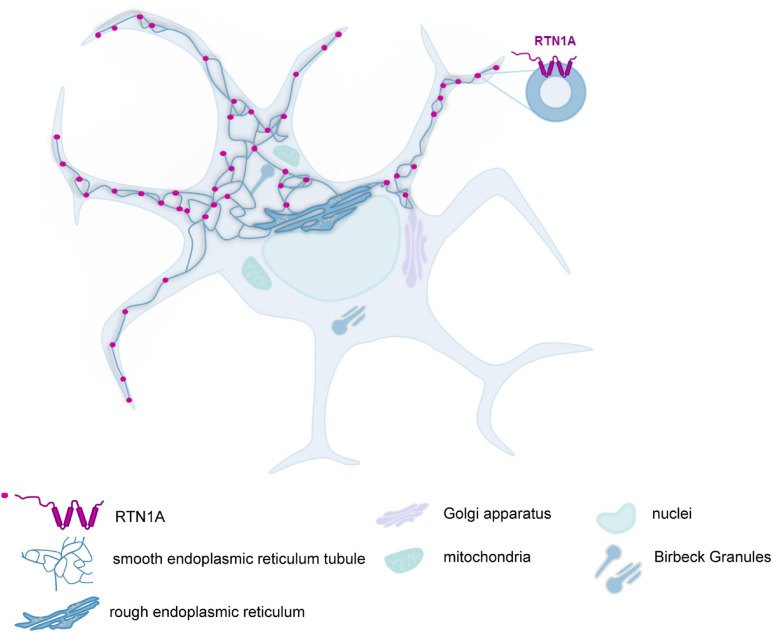
RTN1A protein is enriched in the dendrites of LCs in mouse skin and DCs in lymphoid organs. Schematic representation of RTN1A topology in the membrane of SER tubules and its distribution in LCs and DCs found in the skin and lymphoid organs.

Here we have analyzed the expression of RTN1A in mouse skin and murine lymphoid organs, an endoplasmic reticulum protein, which was previously considered to be specific for nerve cells. We found that the expression of RTN1A in mouse skin showed considerable differences to that in human skin as it was restricted to LCs but absent from other skin cells. In fetal skin at embryonic day 16.5, RTN1A positive cells were dendritic, CD45 positive but negative for the established LC markers as well as for Thy-1 (data not shown). By contrast, in newborn skin the vast majority of epidermal RTN1A positive cells had acquired expression of CD207 and MHCII showing a continuum from RTN1A^*lo*^ to RTN1A^*hi*^. These data strongly suggest that RTN1A expression represents a marker for LC precursor cells during embryonic development. RTN1A expression by LCs was not altered by migration from the epidermis *in vitro* and by tissue culture up to 72 h. These data are reminiscent of what we have previously reported to happen during the differentiation of MUTZ-3 cells toward LCs, in which RTN1A was also expressed before the emergence of LC-specific markers (Gschwandtner et al., 2018). Whether or not the expression of RTN1A plays an active role for the differentiation of LCs, or for the migration of their precursors into the epidermis, remains to be determined. We observed that in LCs in the skin and in DCs in lymphoid organs *in situ*, RTN1A protein was abundant within their dendrites, which might indicate elongated SER throughout the dendrites of LCs and DCs as illustrated in Figure 6. It has been shown that RTN1A orthologs in yeasts are located in the membrane of SER (Hu et al., 2009). Also Voeltz and colleagues (Voeltz et al., 2006) showed and discussed that in both in yeast and mammalian cells, the reticulon proteins are largely restricted to the tubular ER (SER) and are excluded from the continuous sheets of the nuclear envelope and peripheral ER. RTN1A protein in mouse and human has an ER-retention motif, which indicates its presence in the ER membranes and is thus integrated with ER. Moreover, only the structure of SER allows elongation of the tubules, which has been described previously for dendrites and axons of nerve cells (Ramirez and Couve, 2011). The phenomenon of ER elongation into the dendrites of DCs is not well described yet and there are not many markers that have been established for staining of SER tubules in mammalian cells. As mentioned above, reticulons would be a marker for SER tubules or eventually their interaction partners of the Atlastin protein family, which to our knowledge was never characterized in immune cells and was investigated in neural dendrites in nematode (Liu et al., 2019).

Our observation in LCs is comparable to what has been reported for Purkinje cells where RTN1A protein was also enriched in the dendrites (Shi et al., 2017). Indeed, it has been shown that in mouse brain, ER tubule can be elongated throughout neural dendrites, axons and axon terminals and interact there with plasma membrane (Wu et al., 2017). The phenomenon of ER elongation into the dendrites of LCs/DCs is not well described yet. However, SER tubule network extension into dendrites and cell protrusions enables multiple contact sites of the ER with other organelles and the cell membrane (Wu et al., 2018), which could be also crucial for DCs, as to RTN1A, it was shown to be involved in creating mitochondrial contact sites with ER during inter-organelle communication (Cho et al., 2017).

Since the ER has an important function during processing and presentation of antigens we hypothesize that the presence of RTN1A in LCs and their dendrites might participate in ER-phagy by LC as was reported for other RTN family members in other cell types (Bhaskara et al., 2019). Therefore, in professional APCs such as LCs RTN1A might play a role for appropriate antigen processing and maybe even presentation. However, to prove or disprove this hypothesis further functional experiments will be necessary in the future. In contrast to the abundant and stable RTN1A expression in LCs, we discovered that RTN1A is expressed in cutaneous nerves only in prenatal skin. After birth, RTN1A expression in skin nerves was no longer detectable. Therefore, we hypothesize that RTN1A found in skin nerves before birth participates in axonal outgrowth of nerves into the skin but becomes dispensable after the skin nervous system is fully developed. Some support for this hypothesis comes from findings in *C. elegans* where SER network elongation was found in axons in the distant tips of neurites (Rolls et al., 2002). To support this hypothesis it would be interesting to see whether or not RTN1A expression resumes in regenerating skin nerves upon wounding, however, such investigations are beyond the scope of our present study.

The analysis of primary and secondary lymphoid organs revealed a similar expression pattern of RTNA1A. On the basis of RTN1 RNA expression in immune cells (Genevestigator) as well as of the size and localization of RTN1A^+^ cells in areas specific for the occurrence of APCs we conclude that in lymph nodes, RTN1A is expressed in cDCs and a small population of migrating LCs. In addition to cells which we could clearly identify as, a minor population (less than 2%) of large RTN1A^+^ but CD11c^–^MHCII^–^ cells in the subcapsular sinus of lymph nodes needs to be further investigate. To characterize RTN1A^+^ cells in lymphoid organs by flow cytometry, we employed a similar gating strategy as for skin cells. The summary of RTN1A^+^ cells co-expressing CD45, CD207, and F4/80 in skin and lymphoid organs in adult mice can be found in Table 1. The co-expression pattern and frequency of RTN1A and analyzed markers vary between cell types in investigated organs. We can remark that there is rather no association between RTN1A and CD207 expression as we have observed it in the epidermis (± 70% of double positive cells), in thymus (0%) and inguinal lymph nodes were we found there cell populations (LCs) expressing high levels of CD207, which were RTN1A negative. Similar to skin, T and B cells and pDCs could be excluded from the pool of RTN1A^+^ cells across investigated lymphoid organs and the RTN1A expression by different immune cells is summarized in Table 2. In the thymus, gating on CD45^+^ cells excluded a substantial part of thymic epithelial cells, which because of their size and specific morphology may resemble DCs and were difficult to discriminate with IF imaging *in situ*. In the thymus, we found that RTN1A expression is not associated with expression of APC markers such as CD207 and F4/80 and is thus markedly different from epidermal LCs. By contrast, in lymph nodes and spleen the majority of CD207^+^F4/80^+^ cells co-expressed RTN1A like LCs in the skin. Similar to our observation in epidermal LCs, we observed an enrichment of RTN1A expression in the dendrites of positive cells also in thymus, lymph nodes and spleen. Our results demonstrate that RTN1A expression outside the nervous system is not tissue- but cell type-specific within the skin and lymphoid organs and is confined to LCs and DCs. The finding that during embryogenesis RTN1A expression occurs before the emergence of established LC markers will help in the future to monitor the fate of LC precursors in the skin. The fact that RTN1A is an integral protein of ER and the SER and our finding of its abundance within the dendrites of LCs and DCs suggests potential involvement in processed specific for these cell types i.e., antigen processing and presentation.

**TABLE 2:** RTN1A expression profile of leukocytes in mouse tissues.

Cell types	Skin			Lymphoid organs		
	prenatal	postnatal	adult	thymus	lymph node	spleen
LCs	+	+	+	−	+	−
DCs*	−	−	−	+	+	+
T cells	−	−	−	−	−	−
B cells	−	−	−	−	−	−

*pDCs did not express RTN1A in the skin and lymphoid organs; + = co-expression; − = no co-expression; This summary is based on data of IF images and flow cytometry analyses.

## Materials and Methods

### Mouse Tissues

Prenatal and postnatal studies with wild type C57BL/6 mice were performed as described previously (Elbe et al., 1989). Briefly, fetuses were obtained from timed pregnancies, taking the appearance of vaginal plugs as day 0 of gestation. The day of birth was designated as day 0 of postnatal life. After birth, examined time points were determined as postnatal days (pd). For all expression kinetic experiments, back skin was used. Adult mice (males, 6–16 weeks), were used for the preparation of skin [ear skin (IF); back skin (flow cytometry)] and lymphoid organs such as thymus, inguinal lymph nodes and spleen. Animal experimental procedures were approved by the Animal Experimental Ethics Committee of the Medical University of Vienna and the Austrian Federal Ministry of Science and Research (GZ: BMWFW-66.009/0199-WF/II/3b/2014). Individual mouse experiments were performed in Germany and were approved by the local authorities of the German State North Rhine-Westphalia, Germany (LANUV, permission number 50065A4).

### Human Skin

Abdominal skin samples from anonymous healthy female and male donors (age range: 20–67 years) were obtained during plastic surgery procedures. The study was approved by the ethics committee of the Medical University of Vienna (vote number: 2011/1149) and was conducted in accordance with the principles of the Declaration of Helsinki. Written informed consent was obtained from all participants.

### Sequence Alignments

Protein sequences were retrieved from the GenBank database of the National Center for Biotechnology Information (NCBI)^1^ (Altschul et al., 1990). Gene and protein references can be found in Supplementary Figure 1 and Supplementary Table 1. Alignments were performed with the Multiple sequence alignment tool developed by Florence Corpet^2^ (Corpet, 1988).

### Epidermal Single Cell Transcriptome Analysis

Single cell transcriptome analysis was performed using a publicly accessible tool created by Joost and colleagues^3^ (Joost et al., 2016).

### Cell Isolation Procedures

Upon shaving, pieces of back skin, were floated and incubated on 2.4 U/ml Dispase II (Roche Diagnostics) diluted in RPMI 1640 medium (Gibco, Thermo Fisher Scientific; 40 min, 37°C). Subsequently, epidermis was separated from the dermis and incubated in 0.05% trypsin in EDTA (Gibco; Thermo Fisher Scientific; 3 min, 37°C), washed with phosphate buffered saline (PBS; Gibco, Thermo Fisher Scientific) and filtered through a 40 μm mesh. The dermis was digested with 1.6 U/ml collagenase P and 0.05 U/ml DNase I (both from Sigma-Aldrich; 1.5 h, 37°C) in Dulbecco’s Modified Eagle Medium, washed with PBS and filtered through 70 and then 40 μm mesh. Lymphoid organs were pressed through a 40 μm mesh and washed with PBS. Spleens were additionally incubated with hemolysis buffer (3 min, 4°C). Cells were used for flow cytometry staining and/or immunofluorescent imaging.

### Cell Migration Assay

Epidermal sheets from mouse ears were floated on RPMI 1640 medium supplemented with 10% fetal bovine serum and 1% penicillin-streptomycin (Gibco, Thermo Fisher Scientific) for 24 and 72 h. Emigrated LCs were analyzed using flow cytometry and immunofluorescent imaging.

### Flow Cytometry

Single cell suspensions from processed tissues were washed with PBS and stained with fixable viability dye eFluor 450 (eBioscience) for exclusion of dead cells. Next, cells were stained with labeled antibodies for cell surface markers (20 min, 4°C). Thereafter, cells were washed, fixed (IC Fixation Buffer; eBioscience) and permeabilized (permeabilization buffer; eBioscience) according to manufacturer’s instructions. Intracellular staining was performed using an αRTN1A antibody (clone mon162, abcam; 20 min, 4°C), followed by incubation with a fluorescently labeled secondary antibody. Sample analysis was performed on a FACS Verse flow cytometer with the BD Suite software v1.0.5.3841 (BD Biosciences). Data analysis was done using FlowJo v10.6.1 software (FlowJo^TM^ Software, BD Biosciences).

### Immunofluorescence Staining

Tissues were fixed in 7.5% formaldehyde and embedded in paraffin. Deparaffinized (100% xylol, 70% ethanol) samples were incubated in antigen retrieval buffer (Dako), washed with PBS and incubated with antibodies (overnight, 4°C) and with a secondary antibody on the next day (1 h, room temperature). 4’,6-Diamidino-2-phenylindole dihydrochloride (DAPI; BD Biosciences) was used for nuclear staining. All antibodies and reagents used in this study are listed in Supplementary Table 2.

Langerhans cells emigrated from epidermal ear sheets were collected after 72 h, washed with PBS and placed on poly-L-lysine-coated adhesion slides, fixed with acetone (10 min, 4°C) and stained as described above. All images were taken with an Olympus AX70 microscope with imaging software MetaMorph (Molecular Devices).

As separation of skin compartments is impossible at embryonic day (E) 16.5, whole skin sheets were fixed with ice cold acetone for 10 min, washed with PBS and incubated with antibodies (overnight, 4°C) essentially as described (Elbe et al., 1989). At later time points (E18.5, postnatal days (pd) 1–7, and adults), epidermal sheets were separated from the dermis with 3.8% ammonium-thiocyanate, fixed and stained as described above. For IF imaging of prenatal and postnatal skin, confocal LSM 500 Microscope (Carl Zeiss) with Zen software (Carl Zeiss) was used.

### Quantification of LCs Expressing RTN1A and MHCII in Postnatal Epidermal Sheets

The number of LCs expressing RTN1A and/or MHCII in epidermal sheets from back skin has been enumerated as follows: mean of positive cells from 2 fields per donor (*n* = 3) has been shown as mean (SEM) LC number/0.25 mm^2^.

### Immunohistochemistry Staining

Immunohistochemistry staining was performed essentially as described (Schuster et al., 2014). Paraffin-embedded tissue samples were processed and stained with an αRTN1A antibody (polyclonal anti-RTN1) (Supplementary Table 2). After overnight incubation, the samples were washed and incubated with Strept ABC HRP (DAKO) (30 min, room temperature). Images were taken with an Olympus AX70 microscope with imaging software MetaMorph (Molecular Devices).

### Genevestigator

RTN1 RNA expression levels in immune cells of lymphoid organs in adult mice were analyzed using Genevestigator software^4^ (Hruz et al., 2008). Gene expression values were calculated using standard normalization methods facilitating comparison between different experiments and various commercial microarray platforms. Our analysis was based on the ImmGen Project (Microarray Phase 1-Affymetrix 1.0 ST MuGene arrays-series GSE15907). In brief, primary cells from multiple immune cell lineages were isolated *ex vivo* from adult wild type C57BL/6 male mice and sorted up to 99% purity. RNA was extracted from cells, amplified and hybridized to Affymetrix 1.0 ST MuGene arrays. Details regarding the FACS sorting panel of immune cells and experiment specifications such as RTN1 gene expression values, mouse age and repetition is summarized in Supplementary Table 3.

### Statistical Analysis

Statistical analysis has been performed using GraphPad Prism 7 software. One-way ANOVA or 2-way ANOVA, multiple comparisons (Tukey’s multiple comparisons test) has been used to analyze the significance of data. *P*-values: ns – *p* > 0.05, ^∗^*p* ≤ 0.05, ^∗∗^*p* ≤ 0.01, ^∗∗∗^*p* ≤ 0.001, ^****^p ≤ 0.0001. Number of experiments reflects independent experiments with different donors.

## Data Availability Statement

Raw data supporting the conclusions of this article will be made available by authors, without undue reservation.

## Ethics Statement

The animal study was reviewed and approved by the Animal Experimental Ethics Committee of the Medical University of Vienna and the Austrian Federal Ministry of Science and Research and by the local authorities of the German State North Rhine-Westphalia, Germany. Experiments with human skin were approved by the ethics committee of the Medical University of Vienna.

## Author Contributions

MC, ET, and AE-B designed experiments. MC, KK, MB, and AE-B conducted and analyzed experiments. MH, KS, and MZ provided important resources and support. MC and AE-B wrote the manuscript. ET edited the manuscript. AE-B secured funding and supervised the project. All authors had final approval of the submitted version.

## Conflict of Interest

The authors declare that the research was conducted in the absence of any commercial or financial relationships that could be construed as a potential conflict of interest.

## Footnotes

1 http://www.ncbi.nlm.nih.gov/

2 http://multalin.toulouse.inra.fr/multalin/multalin.html

3 http://linnarssonlab.org/epidermis

4 https://genevestigator.com/
