# Supplementary material for: Distinct Distribution of RTN1A in Immune Cells in Mouse Skin and Lymphoid Organs
Source: Front Cell Dev Biol. 2021 Jan 15;8:608876. doi: 10.3389/fcell.2020.608876 (PMC7853085; doi:10.3389/fcell.2020.608876)
Supplement: Supplementary file 1 [file Data_Sheet_1.zip › M.A.Cichon et al_ manuscript_appendix.docx]

**Appendix**

**Supplementary materials**

**Distinct distribution of RTN1A in immune cells in mouse skin and lymphoid organs**

M.A. Cichoń^1^, K. Klas^1#^, M. Buchberger^1^, M. Hammer^2^, K. Seré^3,4^, M. Zenke^3,4^, E. Tschachler^1^, A. Elbe-Bürger^1^*

^1^Department of Dermatology, Medical University of Vienna, Waehringer Guertel 18-20, 1090 Vienna, Austria

^2^Institute of Cancer Research, Medical University of Vienna, Borschkegasse 8a, 1090 Vienna, Austria

^3^Department of Cell Biology, Institute for Biomedical Engineering, RWTH Aachen University Medical School, Pauwelsstrasse 30, Aachen, Germany

^4^Helmholz Institute for Biomedical Engineering, RWTH Aachen University, Pauwelsstrasse 20, Aachen, Germany

***Corresponding author**

Prof. Adelheid Elbe-Bürger, adelheid.elbe-buerger@meduniwien.ac.at

Medical University of Vienna, Department of Dermatology, Vienna General Hospital

Waehringer Guertel 18-20, 1090 Vienna, Austria

^#^Current address

K. Klas, MSc, Laboratory for Cardiac and Thoracic Diagnosis, Regeneration and Applied Immunology, Division of Thoracic Surgery, Medical University of Vienna, Vienna, Austria

**Figure S1 Comparative analysis of RTN1A protein sequences in human and mouse**

(A) Protein sequence alinement shows high RTN1A sequence homology between these two species. Red underline signifies binding sites for two different αRTN1A antibodies used in this study. Blue boxes indicate the reticulon homology domain (RHD). Protein ref. NP_066959.1, NP_703187.2.

(B) Comparison of RTN1A and RTN1C protein sequences between human and mouse. Red font indicates the reticulon homology domain (RHD) and blue font the N-terminal region of RTN1C. Protein ref. RTN1A: NP_066959.1, NP_703187.2., RTN1C: NP_996734.1, NP_001007597.1

**Figure S2 Gene and protein analysis of adult mouse skin cells**

(A) scRNA-seq map (t-SNE plot) shows mouse epidermal cell populations by unbiased clustering.

(B-D) Comparison of RTN1 gene expression in mouse epidermis with LC markers such as MHCII (H2-M2) and CD207.

(E) Expression of the CD3e gene in the DETC cluster represents the negative control.

(F) Representative flow cytometry analysis of RTN1A^+^ cells in the adult mouse dermis. Living singlets are gated on RTN1A and CD45 as well as MHCII. n=3.

**Figure S3 Presentation of RTN1A^+^ and RTN1A^-^ cells in the brain and lymphoid organs**

(A) Purkinje cells in the adult mouse cerebellum express RTN1A.

(B) T cells do not express RTN1A in indicated primary and secondary lymphoid organs as demonstrated by IF staining of single cell suspensions on adhesion slides and by flow cytometry. n=4.

(C) B cells do not express RTN1A in lymphoid organs as shown by a representative IF *in situ* counterstaining with B220 and CD23. n=3. scale bar: 20 µm.

**Figure S4 RTN1A expression in DC subtypes in inguinal lymph node.**

(A) Percentage of RTN1A^+^ DC subtypes in lymph node. Data were measured via flow cytometry and are shown as standard error of the mean (SEM) from 2 independent experiments. nd- not detectable, n=2.

(B) Representative histogram showing RTN1A expression in indicated DC subtypes.

(C) Representative gating strategy for flow cytometry analysis of lymph node cells. Living singlets were gated as follows: LC/DC: MHCII^+^CD207^+^CD103^-^RTN1A^+^, cDC1CD207^+^: MHCII^+^CD207^+^CD103^+^RTN1A^+^, cDC1 CD207^-^: MHCII^+^CD207^-^CD103^-^RTN1A^+^, cDC2: MHCII^+^CD207^-^CD103^-^XCR1^-^RTN1A^+^, pDC: MHCII^-^CD11c^+^B220^+^RTN1A^+^.

**Table S1** Specifications for human and mouse RTN1A (GenBank accession numbers)

|  | Human | Mouse |
| --- | --- | --- |
| RTN1 gene ID | 6252 | 104001 |
| RTN1A mRNA ID | NM_021136.3 | NM_153457.7 |
| RTN1A protein ID | NP_066959.1 | NP_703187.2 |

**Table S2** Reagents used in the study

| Antibodies | Source | identifier |
| --- | --- | --- |
| Mouse monoclonal anti-RTN1A (clone mon162) | abcam | Cat# ab9274 |
| Rabbit polyclonal anti-RTN1 | Sigma Prestige Antibodies | HPA044249 |
| Rat monoclonal anti-MHCII-FITC | BD Pharmingen | Cat#556999 |
| Rat monoclonal anti-MHCII-BV510 | Biolegend | Cat#107605 |
| Rat monoclonal anti-CD45-PE | Biolegend | Cat#103107 |
| Rat monoclonal anti-CD207-FITC/APC (clone 929F3.01) | Dendritics | Cat#DDX0362 |
| Mouse monoclonal anti-CD207-APC(clone 4C7) | BioLegend | Cat#144205 |
| Rat monoclonal anti-F4/80 | ThermoFisher | Cat#13-4801-82 |
| Rat monoclonal anti-B220 | ThermoFisher | Cat#13-0452-82 |
| Rat monoclonal anti-B220-PerCP-Cy5.5 | eBioscience | Cat#45-0452-82 |
| Hamster monoclonal anti-CD11c | ThermoFisher | Cat#13-0114-82 |
| Rat monoclonal anti-CD23 | ThermoFisher | Cat#13-0232-81 |
| Rat monoclonal anti-CD3-PE-Cy7 | Biolegend | Cat#100219 |
| Rat monoclonal anti-CD3-PE | Biolegend | Cat#100206 |
| Hamster monoclonal anti-CD3e | BD Pharmingen/Bioscience | Cat#553063 |
| Mouse anti-XCR1-BV421 | Biolegend | Cat#148216 |
| Hamster Monoclonal anti-CD103-FITC | eBioscience | Cat#11-1031-81 |
| Rat monoclonal anti-NF-H/-M | DSHB | 2H3 |
| Secondary antibodies | **Source** | **identifier** |
| Alexa Fluor 546 goat anti-mouse IgG | Invitrogen | Cat# A11030 |
| Alexa Fluor 488 goat anti-mouse IgG | Invitrogen | Cat# A11008 |
| Alexa Fluor 546 goat anti-rabbit IgG | Invitrogen | Cat# A11035 |
| Alexa Fluor 488 goat anti-rabbit IgG | Invitrogen | Cat# A32731 |
| Reagents | **Source** | **identifier** |
| DAPI (4′,6-Diamidino-2-phenylindole dihydrochloride) | Sigma-Aldrich | Cat#D9542 |
| Fixable Viability Dye eFluor™ 450 | eBioscience | Cat#65-0863-18 |
| IC Fixation Buffer | eBioscience | Cat#00-8222-49 |
| Permeabilization Buffer (10X) | eBioscience | Cat#00-8333-56 |
| Ultra-LEAF™ Purified anti-mouse CD16/32 Antibody | BioLegend | Cat#101329 |
| Software |  |  |
| GraphPad Prism 7 |  |  |
| FlowJo v10.6.1 |  |  |
| Genevestigator |  |  |
| Adobe illustrator CS6 |  |  |
| Fiji: ImageJ | | |

**Table S3** Specification of affymetrix microarray from Genevestigator: cell types and FACS sorting panels for cell types in lymphoid organs

|  | **cell type** | **FACS sorting panels for immune cells** | **gene expression values*** | | **n** | **age:**  **postnatal week** | | **experiment ID** |
| --- | --- | --- | --- | --- | --- | --- | --- | --- |
| **thymus** | cDCs CD8α^+^ | CD11c^+^/CD8α^+^/CD11b^-^/CD4^-^/MHCII^+^/CD3e^-^/CD19^-^/Gr1^-^/CD161c^-^/ Ter119^-^ | | 12.11 | 3 | 16-63 | MM-00414 | |
|  | cDCs CD8α^-^ | CD11c^+^/CD8α^-^/CD11b^-^/CD4^-^/MHCII^+^/CD3e^-^/CD19^-^/Gr1^-^/CD161c^-^/ Ter119^-^ | | 12.14 | 3 | 16-63 | MM-00414 | |
|  | macrophages | F4/80^+^/N418^-^ | | 9.91 | 2 | 16-63 | MM-00041 | |
|  | T cells CD4^+^ | CD11b^-^/CD11c^-^/CD49b^-^/Gr1^-^/Ter119^-^/CD19^-^/CD4^+^/CD8^-^/TCRβ^hi^/ CD69^+^ | | 9.44 | 3 | 16-63 | MM-00414 | |
|  | T cells CD8^+^ | CD11b^-^/CD11c^-^/CD49b-/Gr1^-^/Ter119^-^/CD19^-^/CD4^-^/ CD8^+^/TCRβ^hi^/ CD69^+^ | | 9.48 | 3 | 16-63 | MM-00414 | |
|  | thymocytes CD4^+^CD8^+^ | CD11b^-^/CD11c^-^/CD49b^-^/Gr1^-^/Ter119^-^/CD19^-^/ CD4^+^/ CD8^int^/TCRβ^hi^ | | 9.44 | 3 | 16-63 | MM-00414 | |
| **lymph node** | cDCs CD4^+^ | CD11c^+^/CD8α^-^/CD11b^+^/CD4^+^/CD45^+^/CD3e^-^/CD19^-^/Gr1^-^/CD161c^-^/ Ter119^-^ | | 13.2 | 3 | 16-63 | MM-00414 | |
|  | cDCs CD8^+^ | CD11c^+^/CD8α^+^/CD11b^-^/CD4^-^/CD45^+^/CD3e^-^/CD19^-^/Gr1^-^/CD161c^-^/ Ter119^-^ | | 11.5 | 3 | 16-63 | MM-00414 | |
|  | pDCs | CD11c^+int^/CD8α^+^/B220^+^/Gr1^+^/CD45^+^/CD3e^-^/ CD19^-^/CD161c^-^/ Ter119^-^ | | 9.39 | 3 | 16-63 | MM-00414 | |
|  | macrophages | CD11b^+^/CD169^+^/F4/80^-^/CD11c^lo^/CD170^-^/CD103^-^/B220^-^/Gr1^-^/ CD90^-^ | | 10.68 | 2 | 16-63 | MM-00414 | |
|  | follicular B cells | Ter119^-^/Ly6c^-^/CD3^-^/CD11b^-^/CD19^+^/IgM^lo^/IgD^+^/B220^+^/CD93^-^/ CD23^+^/CD21/35^+^/CD43^-^/CD24^+^ | | 9.67 | 2 | 16-63 | MM-00414 | |
|  | T cells CD8^+^ naïve | CD11c^-^/CD19^-^/CD49b^-^/Gr1^-^/Ter119^-^/CD4^-^/CD8^+^/TCR^+^/CD25^-^/ CD62L^hi^/CD44^lo^ | | 9.01 | 2 | 16-63 | MM-00414 | |
| **spleen** | cDCs CD8^+^ | CD11c^+^/CD3-/CD8^+^/CD19^-^/B220^-^/CD161c^-^/Ter119^-^ | | 12.76 | 3 | 16-63 | MM-00414 | |
|  | cDCs CD4^+^ | CD11c^+^/CD8α^-^/CD3e^-^/CD11b^+^/CD4^+^/CD45^+^/CD19^-^/Gr1^-^/CD161c^-^ | | 13.18 | 6 | 16-63 | MM-00414 | |
|  | pDCs | CD11c^-int^/CD8α^-^/CD3e^-^/CD45^+^/Gr1^+^/B220^+^/CD19^-^/CD161c^-^/ Ter119^-^ | | 6.64 | 6 | 16-63 | MM-00414 | |
|  | macrophages | F4/80^+^/CD11b^lo^/B220^-^ | | 9.3 | 3 | 16-63 | MM-00414 | |
|  | follicular B cells | Ly6c^-^/CD11b^-^/Gr-1^-^/CD3^-^/Ter119^-^/CD19^+^ /IgM^+^/IgD^+^/B220^+^/CD93^-^/ CD23^+^ | | 9.54 | 2 | 64-255 | MM-00414 | |
|  | marginal zone  B cells | Ly6c^-^/CD11b^-^/Gr-1^-^/CD3^-^/Ter119^-^/CD19^+^/IgM^+^/B220^+^/CD93^-^/ CD23^-^/CD21/35^+^ | | 9.42 | 3 | 64-255 | MM-00414 | |
|  | germinal center B cells | Ter119^-^/Ly6c^-^/CD11b^-^/Gr-1^-^/CD3^-^/CD19^+^/GL7^+^/PNA^+^ | | 9.05 | 3 | 64-255 | MM-00414 | |
| ***** Gene expression values were shown as mean of RTN1 signal intensity (IQR log_2_ scaled); threshold = 9.5.  ^+^ = positive; ^-^ = negative; ^hi^ = high; ^lo^ = low; ^int^ = intermediate | | | | | | | | |
