# Supplementary figures and images for: Distinct Distribution of RTN1A in Immune Cells in Mouse Skin and Lymphoid Organs
Source: Front Cell Dev Biol. 2021 Jan 15;8:608876. doi: 10.3389/fcell.2020.608876 (PMC7853085; doi:10.3389/fcell.2020.608876)

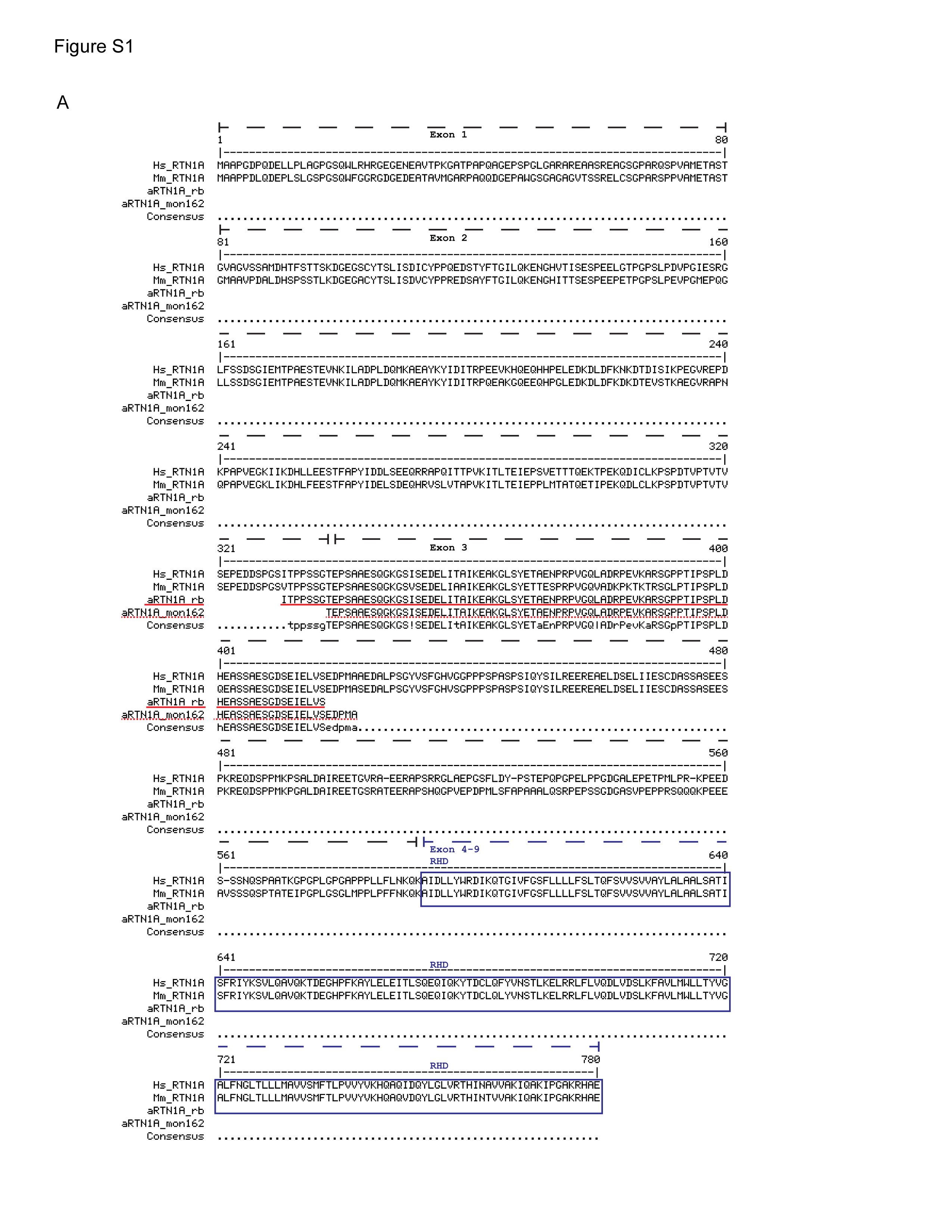

Supplement: Supplementary file 1 [file Data_Sheet_1.zip › Figure S1-A.tif]

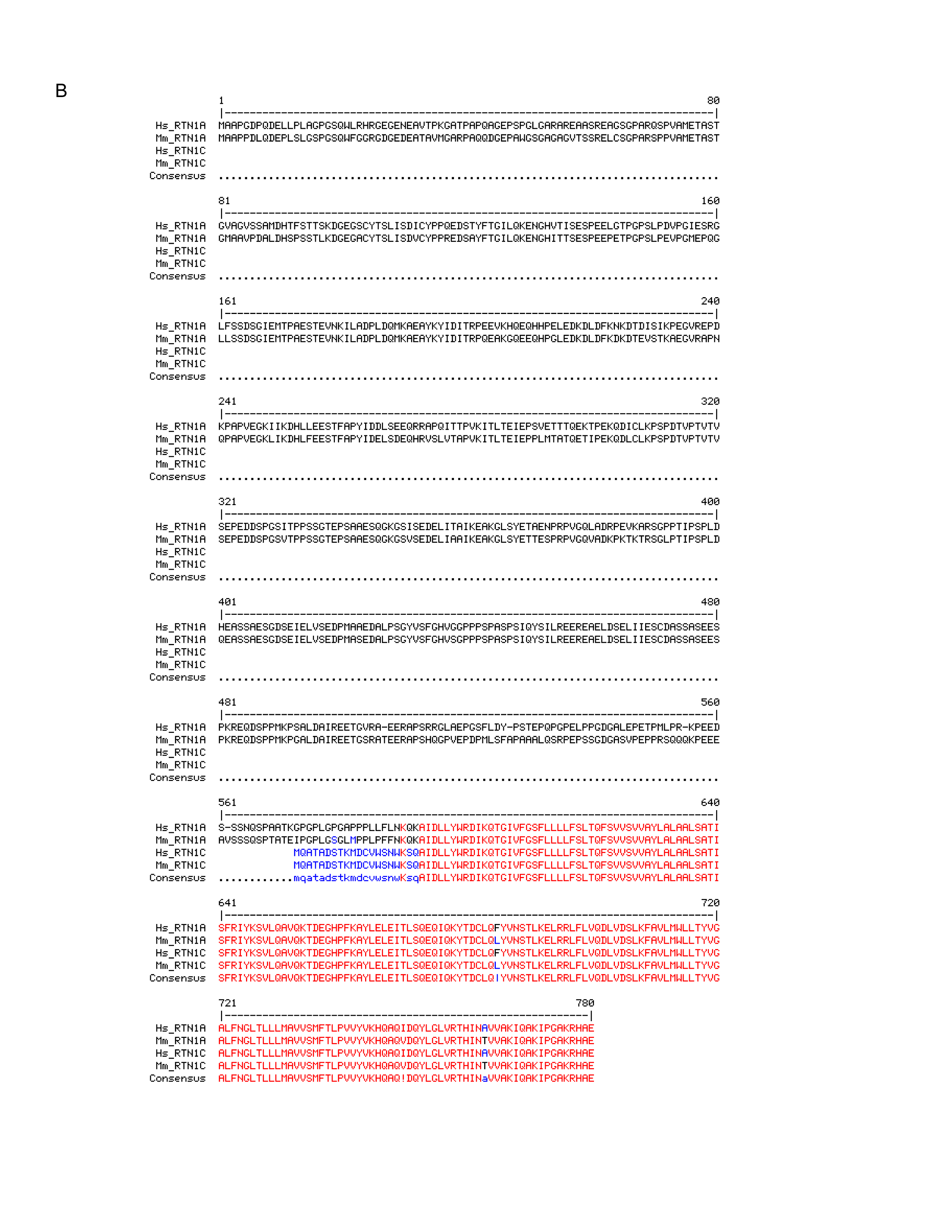

Supplement: Supplementary file 1 [file Data_Sheet_1.zip › Figure S1-B.tif]

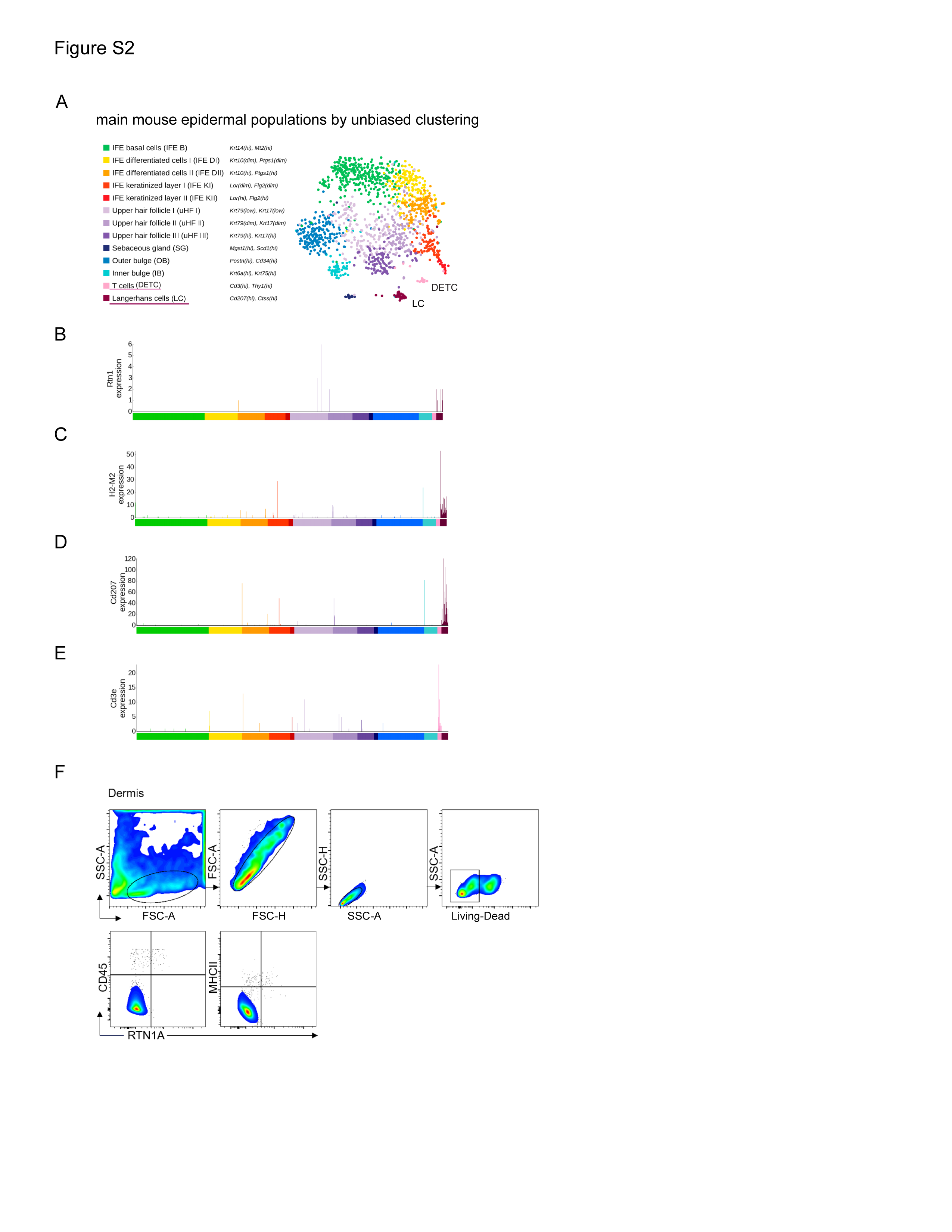

Supplement: Supplementary file 1 [file Data_Sheet_1.zip › Figure S2.tif]

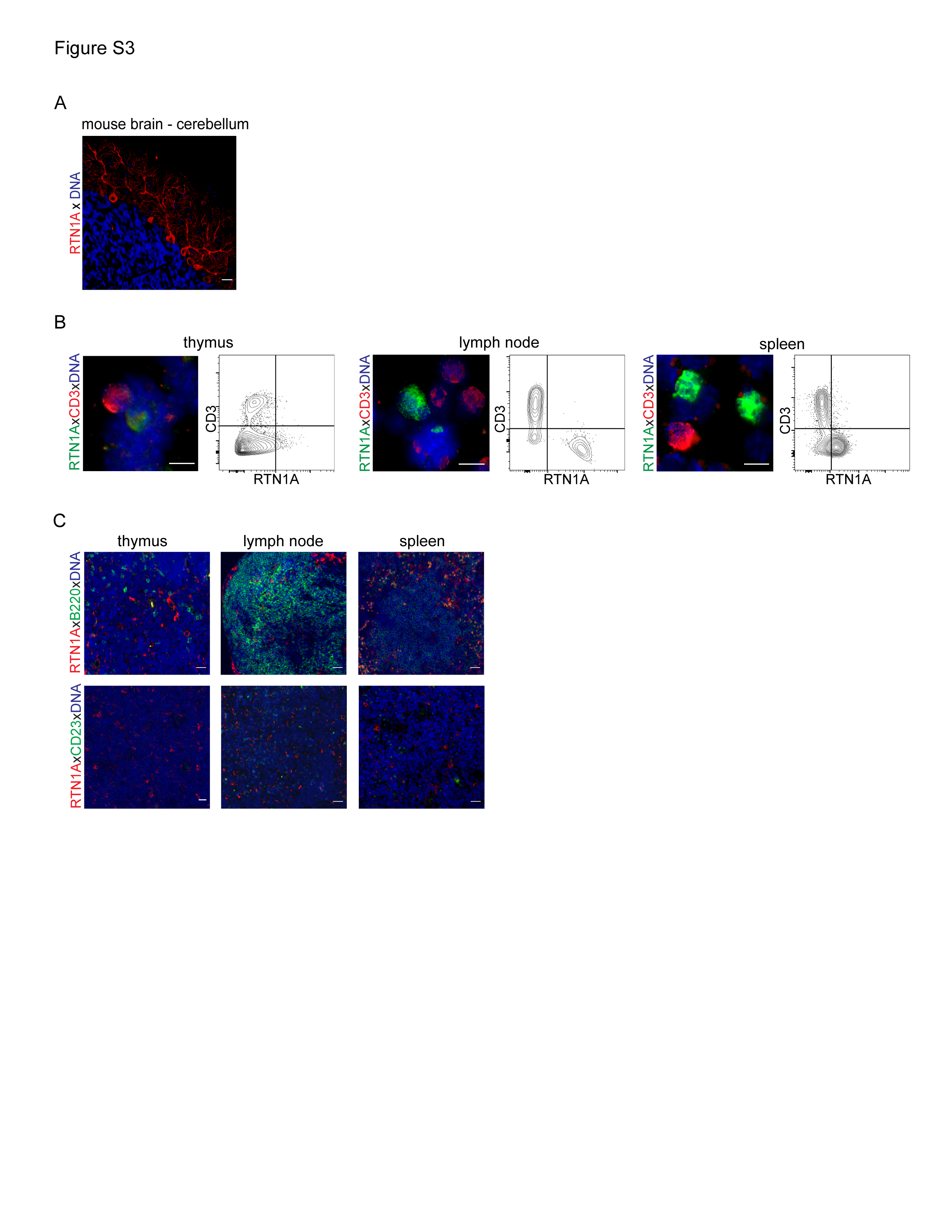

Supplement: Supplementary file 1 [file Data_Sheet_1.zip › Figure S3.tif]

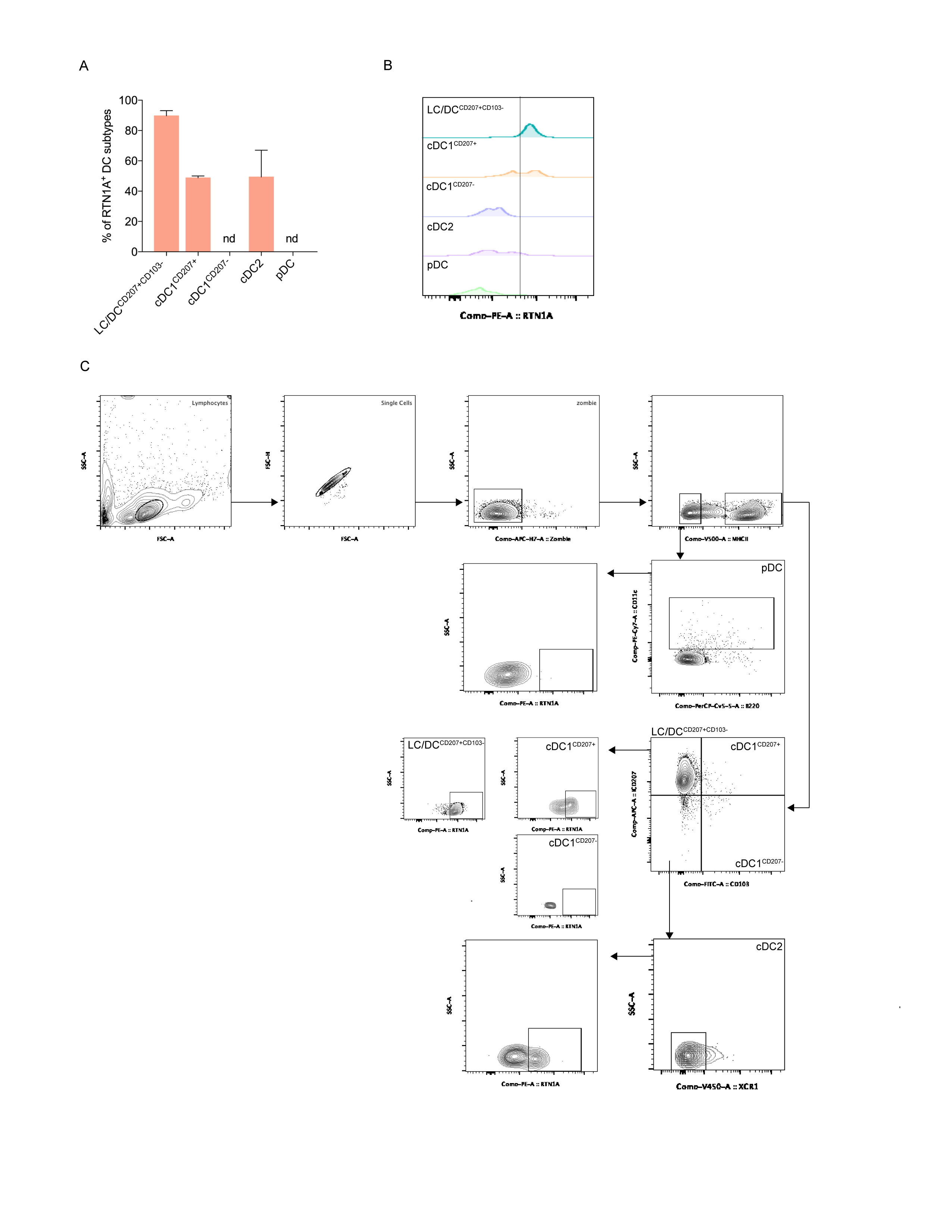

Supplement: Supplementary file 1 [file Data_Sheet_1.zip › Figure S4.tif]
